# A rare presentation of cardiomyopathy associated with Addison's disease: a case report

**DOI:** 10.1093/ehjcr/ytaf455

**Published:** 2025-09-26

**Authors:** Siphy Joseph, Fraser N Witherow, Midhun Paul, Chris Steadman

**Affiliations:** Department of Cardiology, Dorset County Hospital, Williams Avenue, Dorchester DT1 2JY, UK; Cardiologist, Dorset County Hospital, Williams Avenue, Dorchester DT1 2JY, UK; Stroke, Dorset County Hospital, Williams Avenue, Dorchester DT1 2JY, UK; Cardiologist, University Hospital Dorset, Castle Lane East, Bournemouth BH7 7HA, UK

**Keywords:** Addison’s disease, Reversible, Cardiomyopathy, Left ventricular dysfunction, Heart failure, Case report

## Abstract

**Background:**

Cardiomyopathy is an infrequently reported complication of Addison’s disease, which is unique in its reversibility. We report a rare case where cardiomyopathy was the initial manifestation of Addison's disease. This case report explores the pathophysiological association between Addison's disease and heart failure, emphasizing the importance of recognizing this potential link in patients presenting with heart failure.

**Case summary:**

A 39-year-old gentleman presents with classical features of heart failure, which was confirmed by his transthoracic echocardiogram. Addison’s disease was suspected due to hyponatremia, hyperkalaemia, and low cortisol levels. A short synacthen test confirmed the diagnosis, and he was started on dual therapy with hydrocortisone and fludrocortisone. His left ventricular function returned to normal within 5 days of dual therapy.

**Conclusion:**

This case report underscores the critical association between Addison's disease and heart failure, highlighting the importance of considering adrenal insufficiency as a potential underlying factor in cardiac dysfunction. It serves as a reminder of the complex physiological interactions that can cause cardiomyopathy, and further research is vital to improve understanding. Timely recognition and prompt treatment with steroids can lead to rapid reversal of cardiac function.

Learning points

- Cardiomyopathy is a rare but recognized association of Addison’s disease, which can be reversible with dual therapy of glucocorticoids and mineralocorticoids.
- Prompt recognition and initiation of pharmacotherapy are essential for the reversal of symptoms and recovery of cardiac function.
- This case highlights the importance of maintaining a high index of suspicion and conducting a thorough metabolic evaluation in patients presenting with heart failure of unclear etiology.

## Introduction

Addison's disease or primary adrenal insufficiency is a disorder that affects the adrenal glands, causing reduced production of glucocorticoids, mineralocorticoids, and adrenal androgens.^1–8^ Often underdiagnosed due to nonspecific symptoms, it remains potentially fatal but is readily managed with steroid replacement.^1–8^ Autoimmunity accounts for 90% of Addison’s disease cases, with other causes including congenital adrenal hyperplasia, infections such as tuberculosis, and drug-induced forms.^1^ Typical clinical features include fatigue, hyperpigmentation, gastrointestinal symptoms, salt craving, postural hypotension, and muscle weakness or cramps.^1–8^

## Summary figure

**Table:** 

2 weeks before presentation	The patient developed exertional dyspnoea and chest pain
Day 1 admission	Exertional dyspnoea progressed to dyspnoea at rest and chest pain. ECG showed a normal sinus rhythm. Two sets of highly sensitive Troponin I were within the normal range. Blood tests revealed hyponatremia and hyperkalaemia. Chest X-ray was unremarkable
Day 2 admission	TTE showed moderately impaired left ventricular systolic function (EF: 40–45%) with global hypokinesia, non-dilated RV with mildly impaired longitudinal function (TAPSE: 1.3 cm), and normal chamber sizes
	Coronary angiogram showed unobstructed coronary arteries
Day 3	The morning cortisol level was very low, and the endocrinology team was contacted
Day 4	A short synacthen test confirmed Addison's disease, and dual steroid therapy was initiated
Day 7	Symptoms markedly improved, and the patient was discharged, referred for cardiac MRI
Day 9	Cardiac MRI showed normal biventricular size, wall thickness, and systolic function (short-axis ejection fraction of 55% and Simpson's biplane ejection fraction of 61%), with no regional wall motion abnormalities or significant valve disease. The absence of late gadolinium enhancement suggested no evidence of myocardial infarction or fibrosis
Day 17	Repeat TTE showed normal biventricular function

Cardiovascular complications usually described are hypotension, syncope, and arrhythmias.^1–8^ Development of cardiomyopathy is extremely rare.^1–8^ To the best of our knowledge, only eight adult cases of dilated cardiomyopathy associated with Addison’s disease have been reported.^1–8^ We report a unique case of a 39-year-old male presenting with reversible cardiomyopathy during his initial presentation of Addison’s disease.

## Case presentation

A 39-year-old male presented to our institution, with a 2-week history of chest pain and shortness of breath. He also exhibited systemic symptoms, including fatigue, lethargy, myalgia, and impaired concentration. Furthermore, he experienced a brief episode of diarrhoea and vomiting 1 day, occurring 3 days prior to his admission.

His past history includes hypercholesterolaemia, hypertriglyceridaemia and gastroesophageal reflux disease. Additionally, there is a significant maternal family history of ischaemic heart disease and familial dysbetalipoproteinaemia.

Physical examination on admission revealed a systolic blood pressure (BP) of 90 mmHg with a heart rate of 77 beats per minute (bpm). There were no signs of peripheral or pulmonary oedema. A cardiac examination revealed normal heart sounds.

Laboratory analysis revealed hyponatraemia, 125 mmol/L (reference range: 136–145 mmol/L) and hyperkalaemia, 5.3 mmol/L (reference range: 3.5–5.1 mmol/L), but renal function and inflammatory markers were normal. The high-sensitivity troponin I level was measured at <5 ng/L upon admission (reference range: <34 ng/L) and remained consistent when tested again the next day. D-dimer levels were also within normal limits.

**Figure 1:**
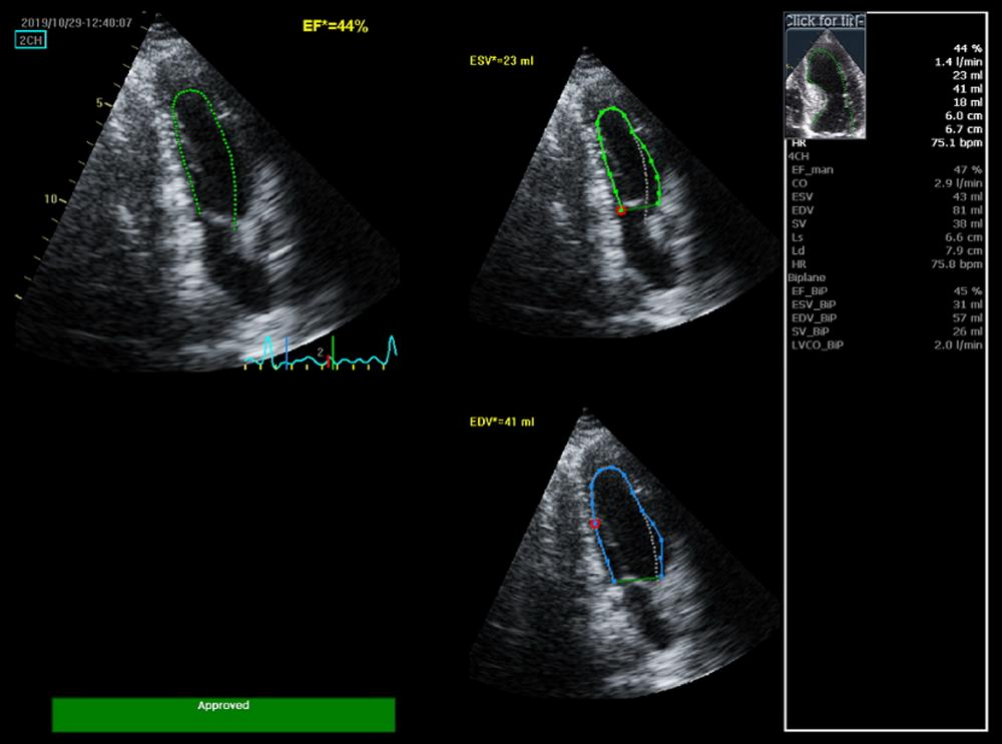
Initial transthoracic echocardiogram showing moderately impaired left ventricular (LV) systolic function, with Simpson's biplane ejection fraction of 44%.

ECG showed normal sinus rhythm at 70 bpm. Chest X-ray revealed clear lung fields. Transthoracic echocardiogram (TTE) demonstrated moderately impaired left ventricular (LV) systolic function (EF: 40–45%) with global hypokinesia, non-dilated right ventricle with mildly reduced function (TAPSE: 1.3 cm), normal chamber sizes and wall thickness, and no significant valvular disease (*Figure 1; Supplementary material online, Videos 1* and *2*).

Due to persistent chest pain, an urgent coronary angiogram was conducted, which revealed no obstruction in the coronary arteries.

Given his abnormal sodium, potassium, and blood pressure levels, a morning cortisol test was performed, revealing markedly low levels, 55 nmol/L (reference range: 133–537 nmol/L), suggestive of Addison’s disease. This was further supported by a short Synacthen test, with cortisol levels consistently between 50 and 60 nmol/L (reference range: >450 nmol/L). Elevated adrenocorticotropic hormone (ACTH) levels of 203 ng/L (reference range: 0–46 ng/L) led to a diagnosis of primary autoimmune adrenalitis.

He was commenced on hydrocortisone (20 mg AM, 10 mg midday, 10 mg evening) and fludrocortisone (100 µg daily). The possibility of pericarditis was considered due to the absence of any signs indicating ischaemic heart disease, despite the absence of biochemical or ECG findings to corroborate this condition. Persistent chest pain was initially managed with colchicine 500 mcg twice daily and ibuprofen 400 mg thrice daily, both of which were promptly discontinued.

Symptoms improved with pharmacotherapy, enabling discharge after six days and scheduling of outpatient Cardiac Magnetic Resonance Imaging (CMR).

**Figure 2:**
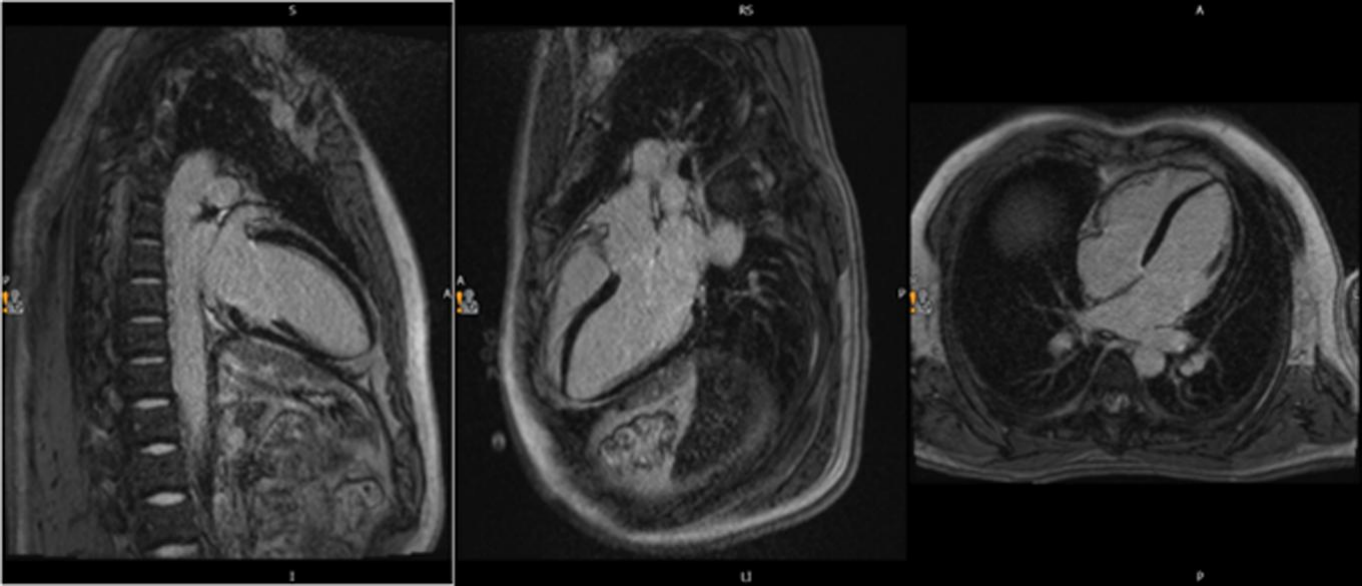
Post-treatment cardiac magnetic resonance (CMR) steady-state free precession (TFLA H2 MAG) cine images in four-chamber (4C), three-chamber (3C), and two-chamber (2C) views. These images demonstrate normal biventricular size with preserved wall thickness. Quantitative tissue characterization performed at the same visit showed normal mapping values at 1.5 T: native T1 = 990 ms, T2 = 49 ms, and extracellular volume (ECV) = ∼25%, consistent with the absence of myocardial oedema or inflammation.

**Figure 3:**
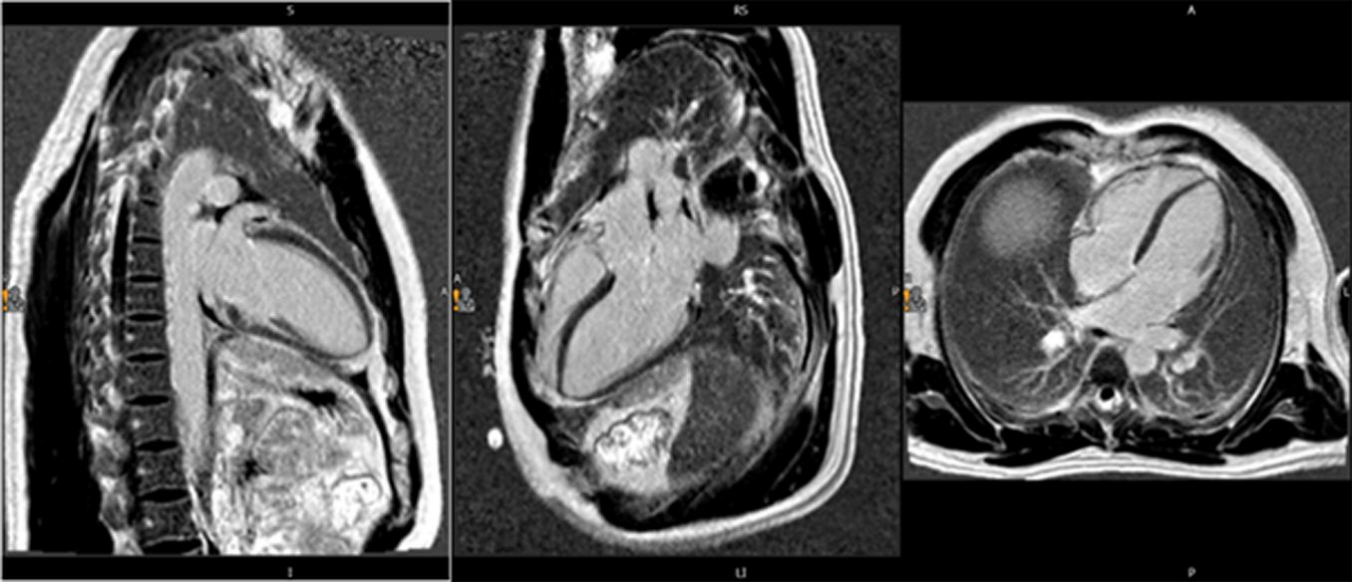
Post-treatment cardiac magnetic resonance (CMR) late gadolinium enhancement (LGE) images acquired with a phase-sensitive inversion recovery (PSIR) sequence in four-chamber (4C), three-chamber (3C), and two-chamber (2C) views. No areas of hyperenhancement are seen, consistent with the absence of focal myocardial fibrosis or scar.

On day 9, following 6 days of steroid therapy, CMR demonstrated normal biventricular dimensions, wall thickness, and systolic function (short-axis: EF 55%, Simpson’s biplane EF: 61%), with no regional wall motion abnormalities or valvular disease. Native T1 (990 ms; ref: 950–1050 ms) and T2 (49 ms; ref: 45–55 ms) values were within normal limits, excluding myocardial oedema or inflammation. Absence of late gadolinium enhancement further indicated no evidence of acute injury or fibrosis *Figures 2* and *3*.

**Figure 4:**
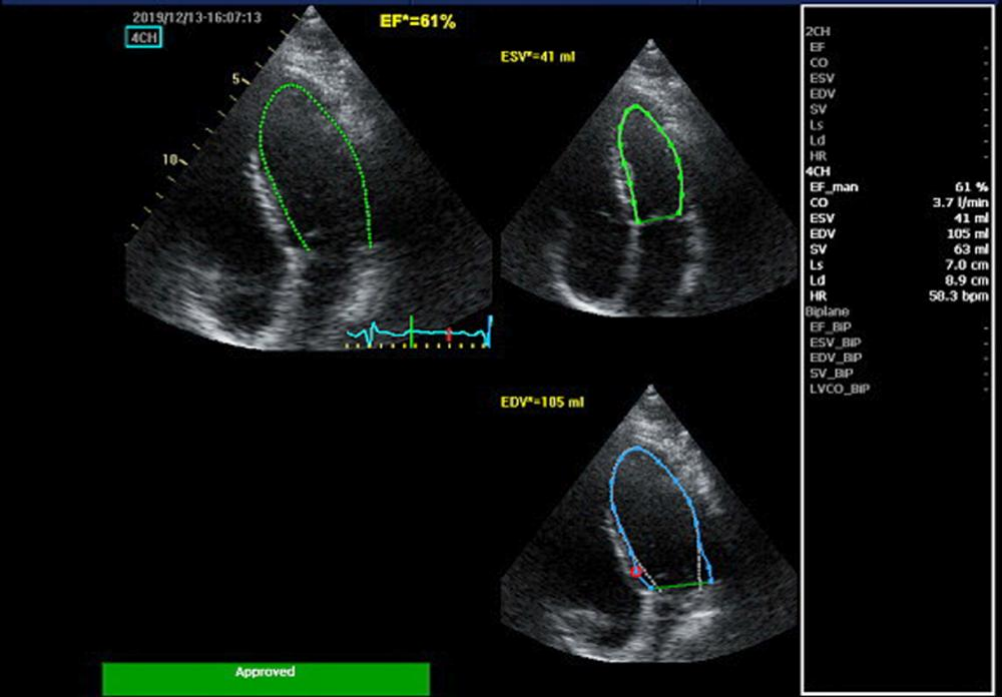
Follow-up transthoracic echocardiogram demonstrating consistently recovered left ventricular function, with a Simpson's biplane ejection fraction of 61%.

His cardiac symptoms resolved within a month, and subsequently repeated TTE after 1 month showed normal biventricular systolic and diastolic function with a biplane ejection fraction of 62% (*Figure 4; Supplementary material online, Videos 3* and *4*).

He was referred to endocrinology for ongoing management of Addison’s disease, with regular folow-up to optimise hormone replacement. One-year follow-up TTE demonstrated normal cardiac function, reflecting a favourable response to treatment.

## Discussion

Primary adrenal insufficiency or Addison’s disease is a rare endocrinopathy with an incidence of approximately 120 cases per million in the world.^1,5^ The first case of new-onset cardiomyopathy associated with Addison’s was described in 1963.^7^ In the majority of the cases, the reduced heart function was reversed with corticosteroid replacement.^1–5^

**Figure 5:**
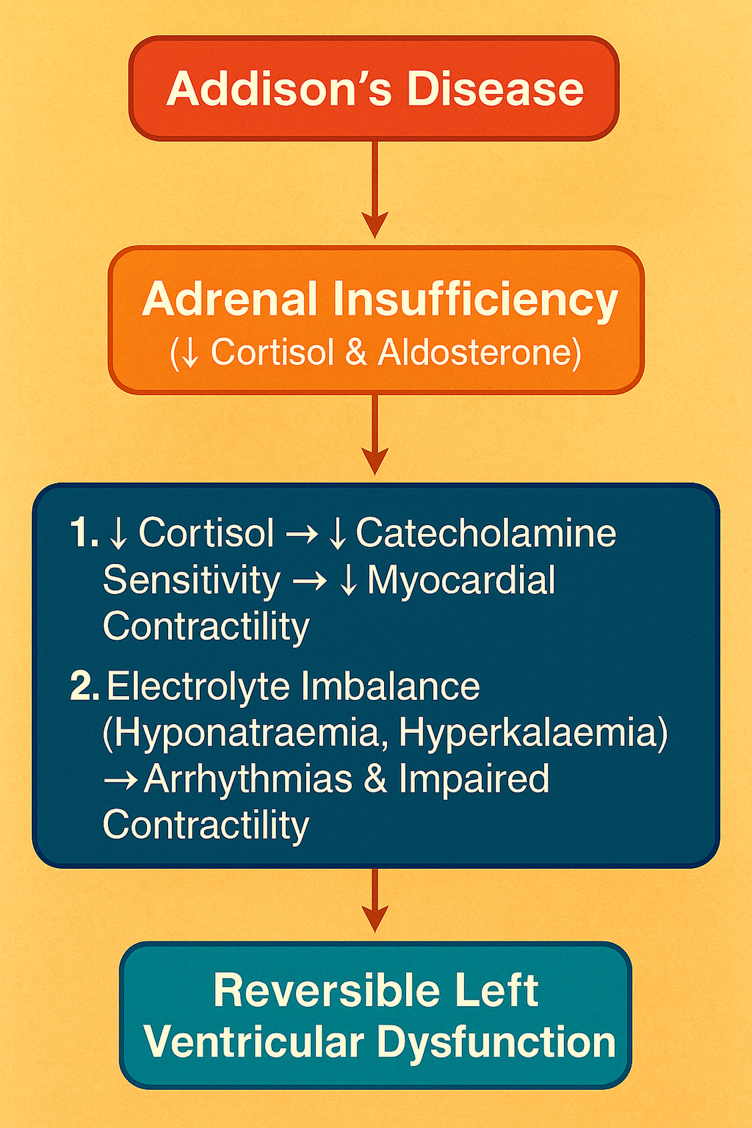
Flow chart on pathophysiological link between Addison’s disease and reversible left ventricular dysfunction via adrenal insufficiency, electrolyte imbalance, reduced catecholamine sensitivity, and hypotension.

The exact pathophysiology of cardiomyopathy associated with Addison’s disease is poorly established, but several factors are likely to contribute, including (i) lack of direct effect of cortical hormones on the myocardium, (ii) dyselectrolaemia—leading to less myocardial excitability, (iii) haemoconcentration indirectly causing poor coronary flow, and (iv) limited nourishment and depleted glycogen stores (*Figure 5*).^1,8^

Animal studies indicate that glucocorticoid deficiency downregulates adrenoceptors, contributing to cardiovascular complications. Specifically, Adrenalectomy in rats caused low calcium uptake by the sarcoplasmic reticulum, resulting in a negative ionotropic effect, which was largely reversed by dexamethasone.^7^ Future research may clarify the mechanisms underlying reversible cardiomyopathy (*Figure 5*).

In our case, cardiomyopathy occurred during the initial presentation of Addison’s disease. Despite biventricular dysfunction and low ejection fraction, the patient showed no peripheral or pulmonary oedema, likely due to the impaired renin-angiotensin-aldosterone system characteristic of Addison’s disease, suggesting that overt signs of heart failure may be masked and reinforcing the need for routine or urgent echocardiography when cardiovascular involvement is suspected.

Although the patient exhibited no biochemical or imaging evidence of pericarditis, NSAIDs were administered briefly to relieve pleuritic chest discomfort, with careful monitoring and prompt discontinuation once the diagnosis was confirmed. This approach highlights the need for caution when using NSAIDs in patients with heart failure, given their potential adverse effects.

In the case presented, the patient exhibited normal high-sensitivity troponin T levels, and a CMR on the ninth day showed no evidence of myocarditis, ruling out other potential causes for the diminished ejection fraction. Additionally, due to the postponement of the CMR, by the time it was conducted, there was a considerable improvement in symptoms, and a return to normal cardiac function was observed.

The rapid reversal of myocardial dysfunction within 6 days of corticosteroid therapy suggests that the myocardium was not primarily affected. Improvement likely resulted from steroids’ direct or indirect effects on electrolyte balance, underscoring the importance of early diagnosis and timely steroid initiation for cardiovascular recovery.^1–5^ Dual therapy with glucocorticoids (hydrocortisone) and mineralocorticoids (fludrocortisone) remains the cornerstone of Addison’s disease management.^1–5,7,8^ However, fludrocortisone may precipitate acute heart failure due to sodium and water retention, necessitating cautious dose titration in patients with cardiomyopathy.

### Similarities and differences in literature

Similar to prior reports, our case exhibits reversible cardiomyopathy associated with Addison’s disease, with normal coronaries, absent myocarditis, and rapid improvement after corticosteroid therapy, though the extent and pattern of ventricular involvement show subtle variations across cases.^1–8^

In contrast to the usual presentation of Addison’s-associated cardiomyopathy, which typically involves left ventricular dilatation and more severe systolic dysfunction, our case demonstrated only mild biventricular impairment without LV dilatation.^1,6^ The transient nature of the dysfunction, with full recovery following hormone replacement, suggests a reversible association rather than a direct causal effect. Furthermore, the absence of pulmonary or peripheral oedema—commonly seen in heart failure—further distinguishes this case.^1,6^

Overall, while the potential association between Addison’s disease and cardiomyopathy is well-established, the precise mechanisms remain unclear. This case adds to the literature by highlighting an atypical presentation, with milder myocardial involvement and absence of left ventricular dilatation, suggesting that other factors beyond classic dilated cardiomyopathy mechanisms may be at play in these patients.

## Conclusion

Cardiomyopathy is rarely associated with Addison’s disease and is not a well-recognized sequela of autoimmune adrenalitis. This case report highlights the potential association between Addison's disease and cardiac dysfunction, highlighting the importance of considering adrenal insufficiency as a possible underlying factor in cardiac dysfunction. While further studies are required to clarify the mechanisms of reversibility, our findings highlight that prompt recognition and initiation of dual corticosteroid therapy are pivotal for recovery. Additionally, this case serves as a reminder of the complex physiological interactions that can exacerbate heart failure and the need for a comprehensive approach to patient care.

## Supplementary Material

ytaf455_Supplementary_Data

## Data Availability

The data underlying this article are available in the article and in its online Supplementary material.

## Lead author biography

Dr Siphy Joseph is an Internal Medicine Trainee, working in Dorset County Hospital of Wessex Deanery. She has an interest in Cardiology.
