# Supplementary figures and images for: A rare presentation of cardiomyopathy associated with Addison's disease: a case report
Source: Eur Heart J Case Rep. 2025 Sep 26;9(11):ytaf455. doi: 10.1093/ehjcr/ytaf455 (PMC12690470; doi:10.1093/ehjcr/ytaf455)

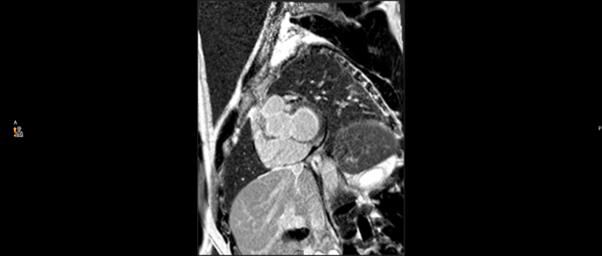

Supplement: ytaf455_Supplementary_Data [file ytaf455_Supplementary_Data.zip › CMR T FLASH2 PSIR SAX.png]

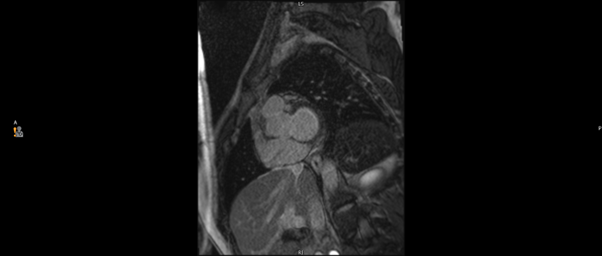

Supplement: ytaf455_Supplementary_Data [file ytaf455_Supplementary_Data.zip › CMR TFLASH2 MAG SAX.png]
